# Supplementary material for: Magnitude of multidrug-resistant and extended-spectrum β-lactamase-producing gram-negative bacteria from tracheal aspirates of intensive care unit patients in Ethiopia
Source: PLoS One. 2025 Jun 24;20(6):e0324199. doi: 10.1371/journal.pone.0324199 (PMC12186978; doi:10.1371/journal.pone.0324199)
Supplement: Attachment — Submitted filename: ZGB point by point responses for reviewers.odt [file pone.0324199.s004.odt]

Areas for Improvement and Recommendations 
1.Introduction
1. Contextual Relevance: While the introduction effectively establishes the global significance of antimicrobial resistance, it lacks sufficient contextualization for Ethiopia. Discuss specific challenges or gaps in surveillance and resistance patterns in the region.
Response: Thank you for your insightful comment. And comments are incorporated. line 95-107 
2. Objectives: Explicitly state the research objectives as testable hypotheses or specific aims to guide the reader.
Response: Thank you for your comment. We revised the manuscript to explicitly state the research objectives as specific aims as follow. The objective of the study was to identify Gram-negative bacterial pathogens from tracheal aspirates, determine the prevalence of MDR, and assess the prevalence of ESBL production among the isolates. Line 34-36
2.Methods
Study Population:
3. Define inclusion/exclusion criteria more clearly, including the rationale for excluding tuberculosis patients and those intubated for less than 48 hours.
Response: Thank you for your valuable feedback. We excluded tuberculosis patients due to the distinct microbiological and clinical characteristics of Mycobacterium tuberculosis, which require specialized diagnostic and management approaches outside the scope of this study.
Patients intubated for less than 48 hours were excluded to align with the standard definition of ventilator-associated pneumonia (VAP), which typically develops after 48 hours of mechanical ventilation. This criterion ensures the study focuses on cases likely related to prolonged intubation, providing more meaningful insights into VAP-associated MDR organisms. Lines 123-130
4. Specify whether demographic or clinical factors (e.g., comorbidities, prior antibiotic use) were considered.
Response: Thank you for your comment. We included both demographic and clinical factors in our analysis. Listed and incorporated in line 132-135
Sample Handling:
5. Provide more details on how sample collection was standardized to minimize contamination. Include information on how samples were transported and processed to ensure timely and accurate results.
Response: Thank you for your valuable comments: incorporated in lines 139-150.
6. Antimicrobial Susceptibility Testing (AST): Justify the selection of the antibiotics tested, particularly for carbapenems and aminoglycosides, and explain their relevance to local prescribing practices. Clarify if and how intermediate susceptibility results were categorized in the analysis.
Response: Thank you for your valuable comments: Carbapenems, ` in particular, were chosen to treat critically ill patients in intensive care units who developed VAP as a result of ESBL and carbapenemase-producing infections, which are common in our region and widely prescribed in our settings as last-resort of treatment. Lines 181-184
Intermediate susceptibility values were considered reduced susceptibility and treated as resistant in the analysis to ensure appropriate reporting and antimicrobial stewardship guidance. Lines173-175
Statistical Analysis:
7. Describe the handling of missing data and whether sensitivity analyses were performed.
Explain the rationale for including variables in the logistic regression model and how multi-collinearity was assessed?
Response: We carefully worked through source documents such as laboratory results and patient files to assess data completeness as well as missing or incorrectly entered information, if any. We made efforts to go back to original records and consult with site data collectors to retrieve missed data.
The variables included in the logistic regression model were selected based on clinical relevance and statistical significance in univariate analyses with p. value less than 0.25 were candidate for multivariate logistic regression analysis. Before interpreting the findings, we calculated the variance inflation factor (VIF) and tolerance to verify the impact of multicollinearity among the independent variables. In multivariate analysis, a P-value less than 0.05 was considered statistically significant. Lines 210-221
Results
8. Data Presentation: 
Provide stratified data to explore variations in MDR/ESBL prevalence by age, sex, or ICU type (e.g., pediatric vs. adult).
Response: Thank you for your insightful comment and we have incorporated and adjusted your suggestion in Table 2. line 247
9. Avoid redundancy by streamlining overlapping information in the text, tables, and figures.
Key Findings:
Highlight the clinical significance of the high resistance rates observed for specific antibiotic classes, particularly carbapenems and cephalosporins.
Response: Thank you very much and we avoid the redundancy results in the paragraphs which are already included in the tables accordingly.
10. Clarify if there were significant trends or outliers in resistance patterns.
Response: Thank you for your valuable comments. However, our study did not focus on analyzing trends in resistance patterns, as this would require a broader scope and a longer study period to assess potential contributing factors. Investigating AMR trends and incidence over time would necessitate longitudinal surveillance, which was beyond the objectives of our current study. However, we highlighted the resistance trend in the country line 381-392
Discussion
Interpretation of Findings:
11. Delve deeper into the potential drivers of high MDR and ESBL prevalence, such as local antibiotic prescribing practices, infection control gaps, or environmental factors.
Response: Thank you for your thoughtful feedback. However, determining the exact factors contributing to MDR and ESBL prevalence was outside the scope of our research. However, we recognized in the introduction the importance of local antimicrobial prescribing practices, particularly the widespread use of antibiotics without a prescription, which is a major concern in Horn of Africa countries. A deeper look into infection control gaps and environmental factors would need a larger study design, including epidemiological surveillance and antimicrobial stewardship evaluations.
12. Compare findings with a broader range of studies, particularly those from similar healthcare settings in sub-Saharan Africa.
Response: we tried to search similar studies however, there is no similar studies conducted specifically from tracheal aspirates, however, we included few studies about the incidence of infection in VAP.
13. Discuss the implications of the findings for empirical treatment guidelines, particularly in resource-limited settings where access to advanced antibiotics may be constrained.
Response: The findings of MDR and ESBL showed a high prevalence, and from our study participants almost all study participants were under empirical treatment. Studies showed a high resistance trend toward antimicrobials including Carbapenem. lines 363-371
Limitations:
14. Acknowledge the exclusion of gram-positive bacterial and fungal pathogens, and explain how this impacts the study's generalizability and discuss potential biases introduced by the cross-sectional design, such as the inability to establish causality. 
Response: Thank you for your comment. We focused on Gram-negative bacteria given their high prevalence and significant role in MDR in our setting. While this limits the study's generalizability to other pathogen groups, it allows for a more detailed analysis of Gram-negative pathogens and their resistance patterns. Furthermore, our study approach captures associations at a particular point in time, which might not accurately represent temporal relationships between variables. Line 396-400
15. Reviewer #4: Suggested changes and queries and comments have been addressed adequately by the authors. Grammatical errors have been corrected.
Response: Thank you for your time and effort in reviewing our manuscript.
16. Reviewer #5: Dear authors,
Thank you for taking time to address our earlier concerns. I have no further comments at this stage.
Response: Thank you for your time and effort in reviewing our manuscript.
17. Reviewer #6: (No Response)
18. Reviewer #7: The manuscript describes the prevalence of MDR organisms obtained in Tracheal cultures in their setting. There is no justification for selecting this specimen or organ system as MDR organisms are also prevalent in other organ systems and specimen types.
Moreover, the study in its present form does not offer a meaningful insight into the management of VAP and / or the reasons for the emergence of MDR organisms in this system.

The authors may do well to have more clinical orientation with an attempt to connect the microbiology and the clinical and radiological evidence of pneumonia.
Insight into other organ systems should be important to better define the prevalence and magnitude of the problem of MDR & ESBL producing GNB in hospital practice
Response: Thank you for your insightful comments. The focus on tracheal aspirate cultures was chosen due to its critical role in diagnosing ventilator-associated pneumonia (VAP), a significant concern in our clinical setting. While MDR organisms are indeed prevalent in other specimen types, tracheal cultures provide a direct and reliable indication of lower respiratory tract infections in intubated patients. And this study will help as a baseline to expand the research surveillance in our facilities. LINE 139-141.
We strongly believe that linking microbiological data to clinical and radiographic evidence of pneumonia is critical. Integrating microbiological data with clinical features and radiological findings helps to gain a better understanding of VAP management and the recent development of MDR pathogens. However, due to resource constraints, and the radiological order was not commonly requested routinely and such data will not be representative for the study participants.
