# Supplementary material for: Magnitude of multidrug-resistant and extended-spectrum β-lactamase-producing gram-negative bacteria from tracheal aspirates of intensive care unit patients in Ethiopia
Source: PLoS One. 2025 Jun 24;20(6):e0324199. doi: 10.1371/journal.pone.0324199 (PMC12186978; doi:10.1371/journal.pone.0324199)
Supplement: Attachment — Submitted filename: ZGB3 point by point response to final 2 submission.odt [file pone.0324199.s005.odt]

Areas for Improvement and Recommendations 
1. Lines 173-175: "Intermediate susceptibility test results were considered as "resistant spp." to ensure appropriate reporting in accordance with antimicrobial stewardship guidance.", this statement needs reference.
Response: Thank you for your insightful comment, as per your suggestion we removed the referenced statement. And the AST results have been reanalyzed. Following this minimal changes in resistance patterns of Acinetobacter spp. and K.pneumonae species in Table 3 and specifically, the high resistance level originally reported for Acinetobacter spp. across all antimicrobial classes—especially cephalosporins—has been updated from 89.6%–96.9% to 86.5%–95.8% (Line 252).
The reason for the previous categorization of intermediate as resistance was due to by considering the patients' critical clinical condition, antimicrobial treatment must precede documented susceptibility results rather than intermediate susceptibility, which only promises optimal clinical outcomes. The trend in our study site is to treat critical patients only with susceptible drugs. Additionally, according to the reference (Centre for Health Protection, Department of Health - Antimicrobial Resistance (AMR) Surveillance in Public Hospitals and Clinics - Hospital Authority AMR Data (2022) the intermediate and resistant isolates were considered or categorized as non-susceptible in the AMR surveillance report. 
2. Line 384: "Typhoid resistance" is an incomplete statement. Typhoid resistance to which antibiotics? this statement also needs reference.
Response: Thank you for your insightful comment. The section has been carefully reviewed and revised accordingly. The necessary edits have been incorporated, and a relevant reference has been added. However, the reference was taken from the WHO report on “Addressing the Challenge of Antimicrobial Resistance in Ethiopia. This doesn’t mention the type of antimicrobial agents”. The antimicrobial resistance level in the causative agent of typhoid fever increased from 10% in 2006 to 87% in 2019 and similarly, E. coli strains showed increased resistance to stronger antimicrobial agents. Line 377-379
